# Efficacy of leupeptin in treating ischemia in a rat hind limb model

**DOI:** 10.14814/phy2.15411

**Published:** 2022-08-03

**Authors:** Mikhail Gurevich, Kari Iocolano, Irene Nozal Martin, Gurtej Singh, Sami U. Khan, Duc T. Bui, Alexander B. Dagum, David E. Komatsu

**Affiliations:** ^1^ Renaissance School of Medicine Stony Brook New York USA; ^2^ Stony Brook University Stony Brook New York USA; ^3^ Division of Plastic and Reconstructive Surgery, Department of Surgery Stony Brook University Hospital Stony Brook New York USA; ^4^ Department of Orthopaedics and Rehabilitation Stony Brook University Hospital New York USA

**Keywords:** calpain, ischemia, leupeptins, tourniquets, vascular system injuries

## Abstract

Prolonged tourniquet use can lead to tissue ischemia and can cause progressive muscle and nerve injuries. Such injuries are accompanied by calpain activation and subsequent Wallerian‐like degeneration. Several known inhibitors, including leupeptin, are known to impede the activity of calpain and associated tissue damage. We hypothesize that employment of leupeptin in a rat model of prolonged hind limb ischemia can mitigate muscle and nerve injuries. Sprague–Dawley rats (*n* = 10) weighing between 300–400 g were employed in this study. Their left hind limbs were subjected to blood flow occlusion for a period of 2‐h using a neonatal blood pressure cuff. Five rats were given twice weekly intramuscular leupeptin injections, while the other five received saline. After 2 weeks, the animals were euthanized, their sciatic nerves and gastrocnemius muscles were harvested, fixed, stained, and analyzed using NIH Image J software. The administration of leupeptin resulted in larger gastrocnemius muscle fiber cross‐sectional areas for the right (non‐tourniquet applied) hindlimb as compared to that treated with the saline (*p* = 0.0110). However, no statistically significant differences were found between these two groups for the injured left hindlimb (*p* = 0.1440). With regards to the sciatic nerve cross‐sectional areas and sciatic functional index, no differences were detected between the leupeptin and control treated groups for both the healthy and injured hindlimbs. This research provides new insights on how to employ leupeptin to inhibit the degenerative effects of calpain and preserve tissues following ischemia resulting from orthopedic or plastic surgery procedures.

## Graphical abstract

This study provides new insights on how to employ leupeptin to inhibit the degenerative effects of calpain and preserve tissues following ischemia resulting from orthopedic or plastic surgery procedures.

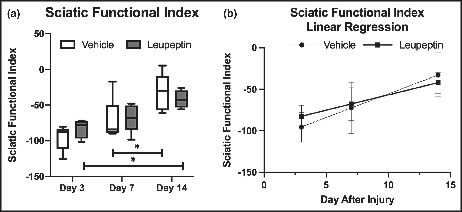

## 1 INTRODUCTION

Ischemia, the interruption of blood flow to an organ, will cause damage to the blood‐deprived organ if prolonged for enough time. A safe duration of ischemia ultimately depends on the organ and tissue being deprived of blood flow. In the case of muscle, nerve, and vascular tissue, a safe time limit of ischemia is somewhere between 1–3 h, but this remains poorly defined (Drew et al., 2015; Pedowitz, 1991). In general, the less time tissues spend under ischemia the better they recover.

Application of surgical tourniquets to the limbs in various settings, such as orthopedic or plastic surgical procedures or during pre‐hospital emergency medical services (EMS) treatment results in tissue ischemia. While tourniquets are beneficial for decreasing blood loss, prolonged tourniquet applications, which are sometimes unavoidable, can lead to tissue damage (Tran et al., 2011). Among vascular, muscle, and nerve tissue, nerve tissue is most prone to tourniquet induced damage (Sharma & Salhotra, 2012).

Currently, there is no solution to prevent tissue injury that arise from ischemia due to prolonged tourniquet use. Such injury to nerves can cause loss of nerve function ranging from neuropraxia, which is reversible, to Wallerian degeneration, which causes varying degrees of irreversible changes (Kilinc et al., 2011; Wang et al., 2015). The main molecules responsible for this process are a family of calcium‐dependent proteases called calpains that are activated during Wallerian degeneration (Ma et al., 2013). In a sciatic nerve rat model, Wallerian‐like nerve degeneration with marked decrease in conduction velocity, and in extreme cases nerve block, is likely driven by calpain (Makitie & Teravainen, 1977). Muscle degeneration from ischemia and reperfusion is also reported to involve calpain activation in mouse models as reported by Luo et al. In addition to nerves and muscles, calpains are expressed in the vascular wall and their activation has been implicated in several vascular inflammatory and degenerative disorders including atherosclerosis, aneurysms, diabetic angio‐ and retinopathy, and fibrotic and proliferative vascular disease (Etwebi et al., 2018). Over‐activation of calpains has been linked to various stress‐induced situations that can cause increased release of calcium ions (Miyazaki & Miyazaki, 2018; Potz et al., 2016a; Yu et al., 2018). However, proper calpain activation is needed for angiogenesis (the normal physiological response to prolonged ischemia) (Zhang et al., 2017). Additionally, calpains are ubiquitous throughout the mammalian body and has actions in a wide range of tissues including brain, eyes, heart, lungs, pancreas, kidneys, vascular system, and skeletal muscle (Potz et al., 2016b). There are many known calpain isoforms including μ‐, m‐ and several n‐calpains (more than 25). Some of these calpains are “ubiquitous” owing to their presence in all cells of vertebrates such as μ‐, m‐, calpains 10 and 13. On the other hand, “tissue‐specific” calpains are found in only certain tissues and cells such as calpain‐6 (placenta). As our focus is on the rat hind limb muscles, calpain‐3 is likely involved, as well as μ‐ and m‐calpains (Kanzaki et al., 2014; Neuhof & Neuhof, 2014; Pandurangan & Hwang, 2012).

A variety of molecules are known to inhibit calpain. Administration of a calpain inhibitor (alpha‐mercaptoacrylic acid derivative PD150606 [3‐(4‐iodophenyl)‐2‐mercapto‐(Z)‐2‐propenoic acid]) before ischemia had tissue protective effects via the mitochondrial apoptotic pathway (Luo et al., 2015). Numerous studies have also shown the efficacy of leupeptin as an inhibitor of calpain by blocking its degradative action on both intact myofibrils and myofibrillar proteins (Malik et al., 1983; Mykles & Skinner, 1983; Sugita et al., 1980). Previously, no studies have investigated the role of calpain in the degenerative changes that occur in muscular and neural tissues of the limbs after prolonged ischemia. In this current study, we investigate the role of leupeptin, which inhibits calpain activity, in preventing muscle and nerve injury in a tourniquet‐induced rat hind limb ischemia model.

## 2 MATERIALS AND METHODS

### 2.1 Induction of hind limb ischemia and Administration of Leupeptin

Ten male Sprague–Dawley rats weighing 300–400 grams were anesthetized with isoflurane anesthesia and placed on a heating pad. Similar number of animals have been used in the literature (Badalamente & Stracher, 2000; Hurst et al., 1984). The animals were then subjected to a two‐hour period of blood flow occlusion in the left hindlimbs at 300 mmHg by means of a neonatal blood pressure cuff (Neonatal #2, Medline) similar to a study previously described (Kauvar et al., 2007). Half of the rats were randomly selected to receive twice weekly intramuscular (gastrocnemii muscles) injections of leupeptin (Sigma Aldrich) at 12 mg/kg in saline starting immediately after tourniquet release. This concentration of leupeptin was chosen based on previous experiments that utilized the same concentration to modulate the process of nerve healing after sciatic nerve transection and repair in the rat (Hurst et al., 1984). The other randomly selected half of the animals received injections of saline vehicle at the same frequency and comparable volumes (100–200 μl) to serve as controls. The investigators were not blinded to these injections. Blood flow occlusion was confirmed by the loss of a pulse (detectible by a pulse oximeter [MouseSTAT from Kent Scientific]) as well as cyanotic discoloration of the limb.

### 2.2 Gait measurements

Three days, 1 week, and 2 weeks after injury, all animals were monitored for gait quality using the sciatic functional index (SFI), as described previously (Wang et al., 2017). The rats were allowed to walk down a 1.8 m long, 150 cm high, 100 cm wide walkway. Their hind paws were inked with black carbon ink (Platinum, Japan) and paper was placed at the bottom of the walkway. SFI was calculated as a function of experimental paw length (EPL), normal paw length (NPL), experimental toes spread (ETS), normal toe spread (NTS), experimental intermediary toes spread (EITS), and normal intermediary toes spread (NITS):

$$\begin{array}{ccc} \mathrm{SFI} & = & -38.3\left(\frac{\mathrm{EPL}-\mathrm{NPL}}{\mathrm{NPL}}\right)+109.5\left(\frac{\mathrm{ETS}-\mathrm{NTS}}{\mathrm{NTS}}\right)\\ & & +13.3\left(\frac{\text{EITS}-\text{NITS}}{\text{NITS}}\right)-8.8 \end{array}$$

### 2.3 Body and gastrocnemii muscle weights measurements

The weights of the rats were measured at day 3, week 1 and week 2. Additionally, upon euthanasia after 2 weeks, the weights of the extracted injured and contralateral healthy gastrocnemii muscles were also measured.

### 2.4 Histology and image analysis

Two weeks after injury, animals were sacrificed, and tissues (gastrocnemii muscles and sciatic nerves) were harvested from the leupeptin administrated and control groups from both hind limbs. All the tissues were fixed in 4% paraformaldehyde solution (Fisher Scientific Company) for 24 h and 5‐micron paraffin‐embedded transverse sections were cut and stained with Hematoxylin (Gill's Hematoxylin III from Poly Scientific) and Eosin dyes (Eosin Phloxine Alcoholic Working Solution from Poly Scientific) using standard histological methods. The stained slides were imaged at 10X magnification using a Nikon Eclipse E800.

### 2.5 Measurements of gastrocnemii muscles areas

Areas of 30 randomly selected gastrocnemii muscle fascicles per cross‐section from five cross‐sections (20 microns apart) per rat were traced manually on ImageJ (NIH) and areas were calculated automatically. This resulted in 150 (30 × 5) fascicles areas per rat. These 150 cross‐sections were used to calculate average area and variability of area (standard deviation) for each animal. Animals were used as biologic replicates to perform statistical comparisons between groups comparing median areas and variability of areas. Comparisons were performed between healthy gastrocnemius muscle fascicles areas, injured gastrocnemius muscle fascicles areas, and injured gastrocnemius muscle fiber areas as a fraction of healthy areas of the same treatment group.

### 2.6 Measurements of sciatic nerve areas

The areas of the nerves for both the left and right hind limbs were calculated for all the 10 rats. Four replicate images per rat per hind limb were used for the calculation of the nerve areas. Each image contained anywhere from 2 to 5 fascicles that together composed the sciatic nerve. The sum of the fascicle areas for each slide was used for analysis.

### 2.7 Statistics

All results are reported as averages with standard deviations in parentheses. Paired comparisons were made using *t*‐tests. For the gait analyses, a linear regression was conducted followed by comparisons between slopes and determination of non‐zero nature of slope. All analyses were performed using Prism 8 (GraphPad) with an alpha of 0.05.

## 3 RESULTS

### 3.1 Rat weights

Rat weights did not differ between groups at the end of the experiment. The vehicle‐treated group had an average weight of 385 g (Tian et al., 2020) and the leupeptin‐treated group had an average weight of 387 g (Hoang et al., 2011) at the end of the two‐week experiment.

### 3.2 Gastrocnemii muscles weights

**FIGURE 1:**
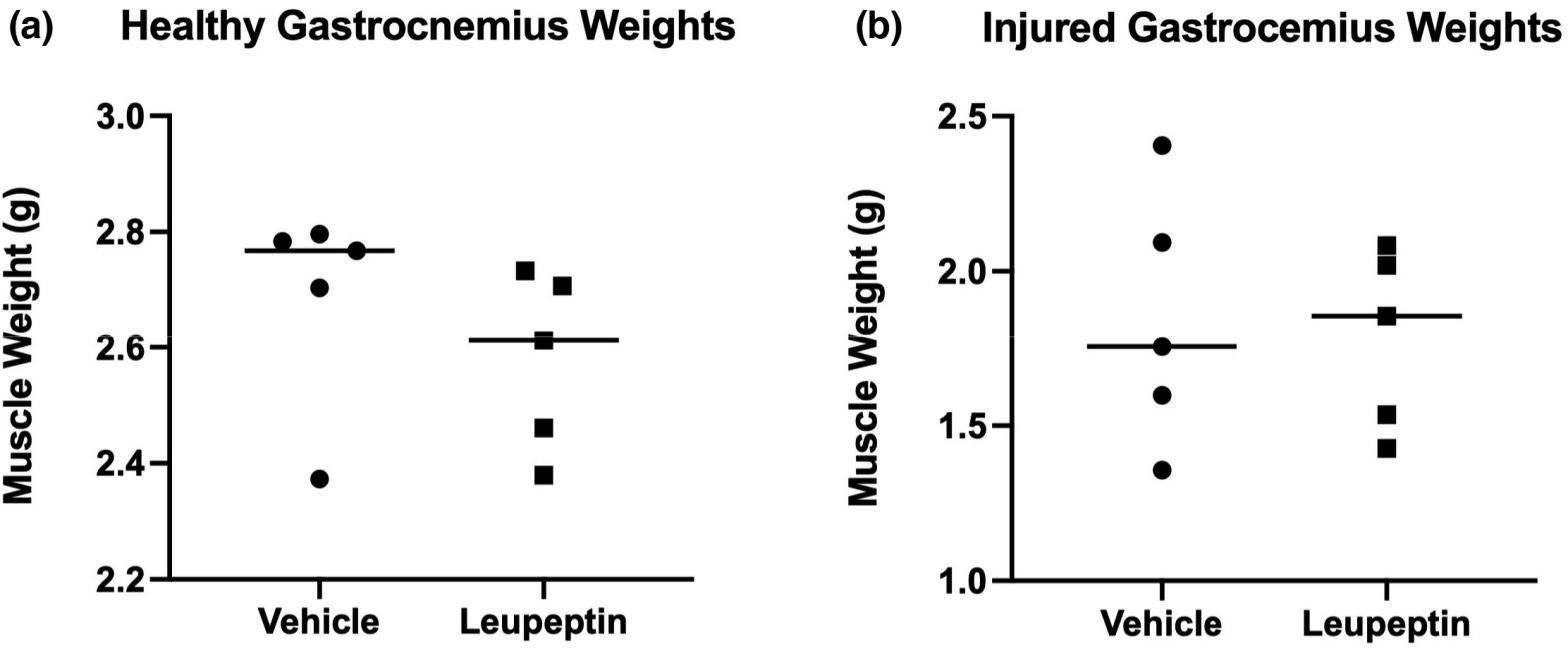
Gastrocnemius weight vs. treatment group shows no difference between treatment groups for healthy (a) or injured (b) gastrocnemius muscle 2 weeks after tourniquet‐induced injury. *N* = 5.

The weights of the gastrocnemii muscles for the right hindlimbs were greater than the left hindlimbs by a factor of ~1.5 for the leupeptin and control groups. However, no significant differences were found between the leupeptin and the control groups (Figure 1).

### 3.3 Gait analysis

**FIGURE 2:**
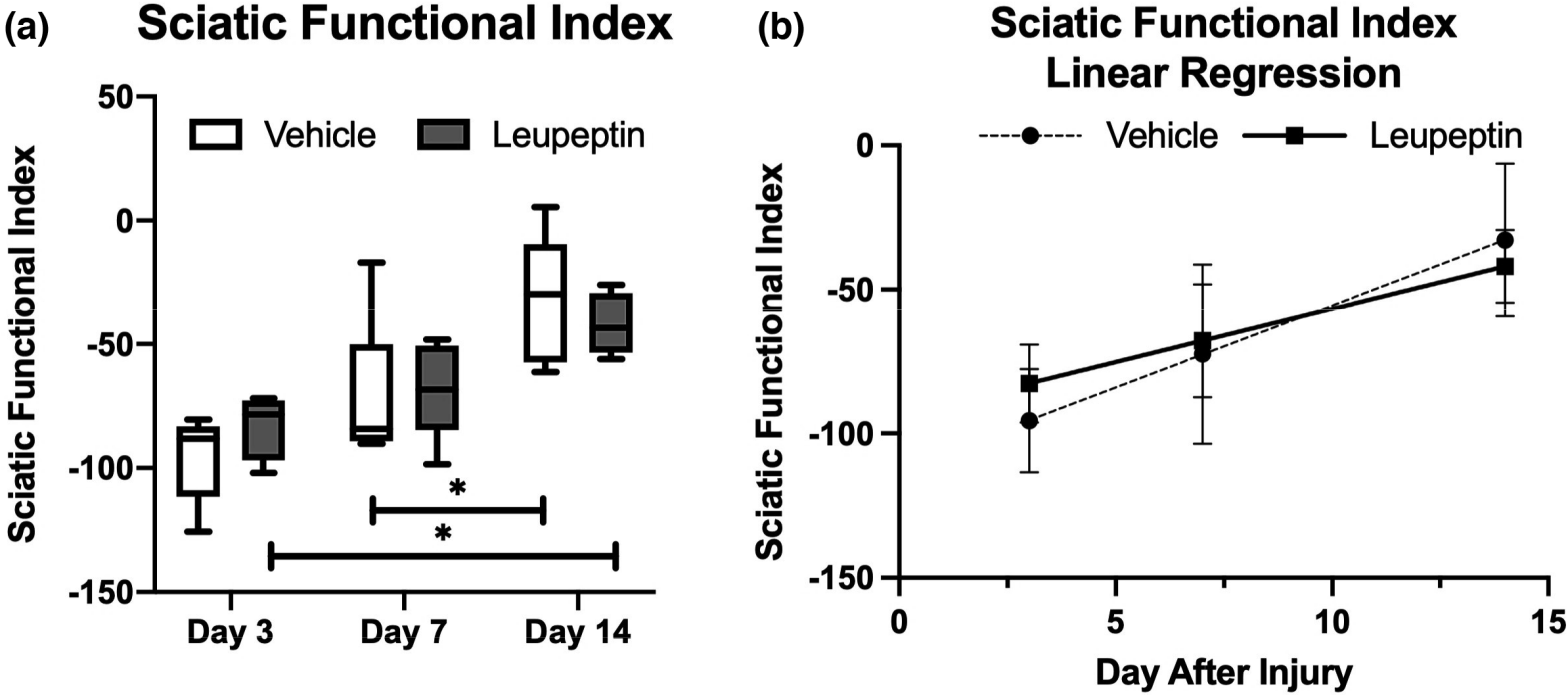
(a) Sciatic functional index as a function of time for both leupeptin‐treated and vehicle‐treated groups shows improvement in SFI over time for both groups, no differences between groups (*n* = 5). Mixed‐effects model with Sidak's multiple comparison test. (b) Linear regressions to SFI over time of both leupeptin‐treated and vehicle‐treated groups shows no differences in slopes.

In both leupeptin‐treated and vehicle‐treated groups, improvements in hind limb functionality were observed over the two‐week period. Leupeptin‐treated animals showed improvement between days three and 14, while vehicle‐treated controls showed improvement between days seven and 14 (Figure 2a). A linear regression of SFI values shows a significantly non‐zero slope for leupeptin‐treated and vehicle‐treated groups, 3.7 and 5.7, respectively (Figure 2b). Both slopes were significantly non‐zero (*p* = 0.0014) and were not dissimilar (*p* = 0.25).

### 3.4 Cross‐sectional areas of gastrocnemii muscles

**FIGURE 3:**
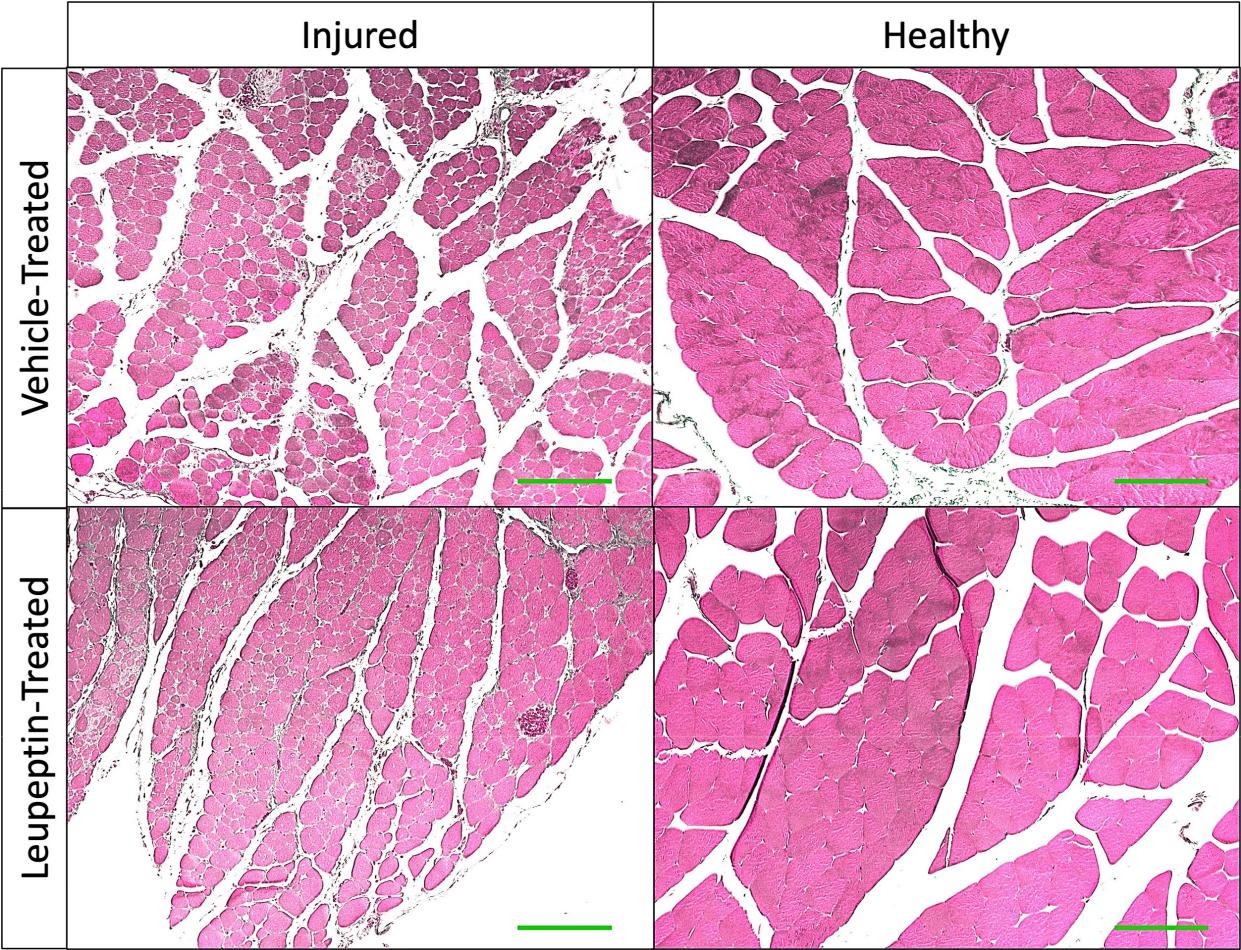
5 μm cross‐sections of injured and contralateral healthy gastrocnemius muscle stained with hematoxylin and eosin for representative animals treated with vehicle or leupeptin 2 weeks after two‐hour blood flow occlusion. Green size‐bars indicate 200 μm.

**FIGURE 4:**
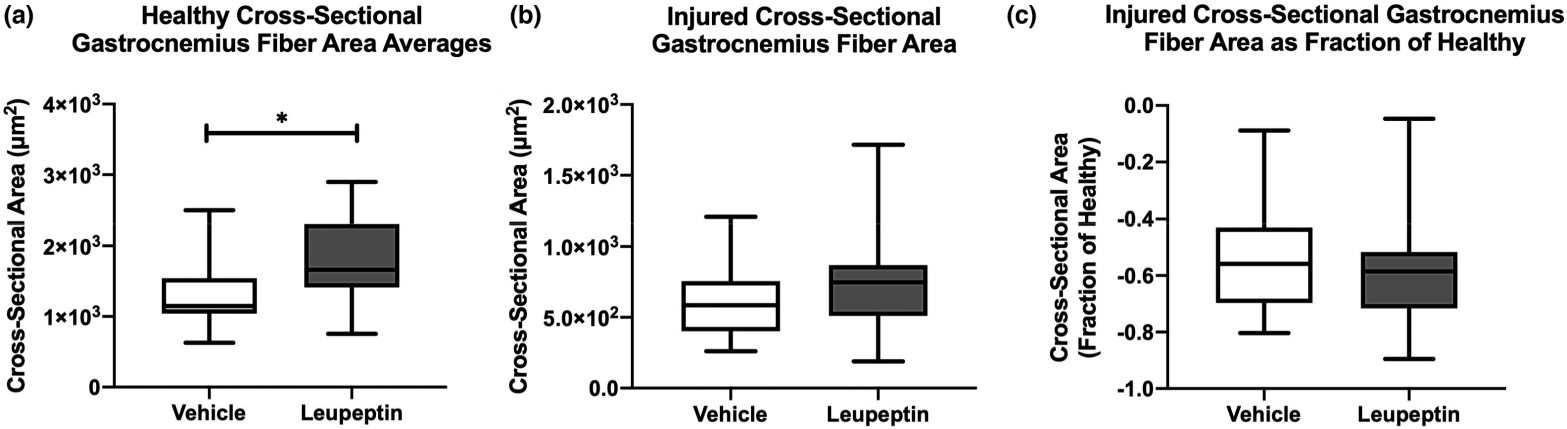
(a) Healthy gastrocnemius muscle fiber cross‐sectional areas are larger after leupeptin treatment than vehicle treatment (*n* = 5). *p* < 0.05 Mann–Whitney test. (b) Injured gastrocnemius muscle fiber cross‐sectional areas are not different after leupeptin treatment or vehicle treatment (*n* = 5) (c) injured gastrocnemius muscle fiber cross‐sectional areas are not different after leupeptin treatment or vehicle treatment after normalization to healthy muscle fiber cross‐sectional area (*n* = 5).

Representative cross‐sections of injured and contralateral healthy gastrocnemius muscle two weeks after injury, with vehicle and leupeptin treatment, respectively, are depicted in Figure 3. The medians of the cross‐sectional areas of healthy gastrocnemius muscle fibers were larger in leupeptin‐treated animals than vehicle‐treated controls, 1663.0 vs. 1147.0 μm (Pedowitz, 1991), respectively (*p* = 0.0110) (Figure 4a). The medians of the cross‐sectional areas of injured gastrocnemius muscle fibers were not different between leupeptin‐treated animals and vehicle‐treated controls, 747.2 vs. 586.1 μm (Pedowitz, 1991), respectively (*p* = 0.1440) (Figure 4b). When normalized as a fraction of contralateral, healthy muscle fiber areas, the medians of the cross‐sectional areas of injured gastrocnemius muscle fibers were not different between leupeptin‐treated animals and vehicle‐treated controls, −0.59 vs. −0.56, respectively, either (*p* = 0.3782) (Figure 4c).

**FIGURE 5:**
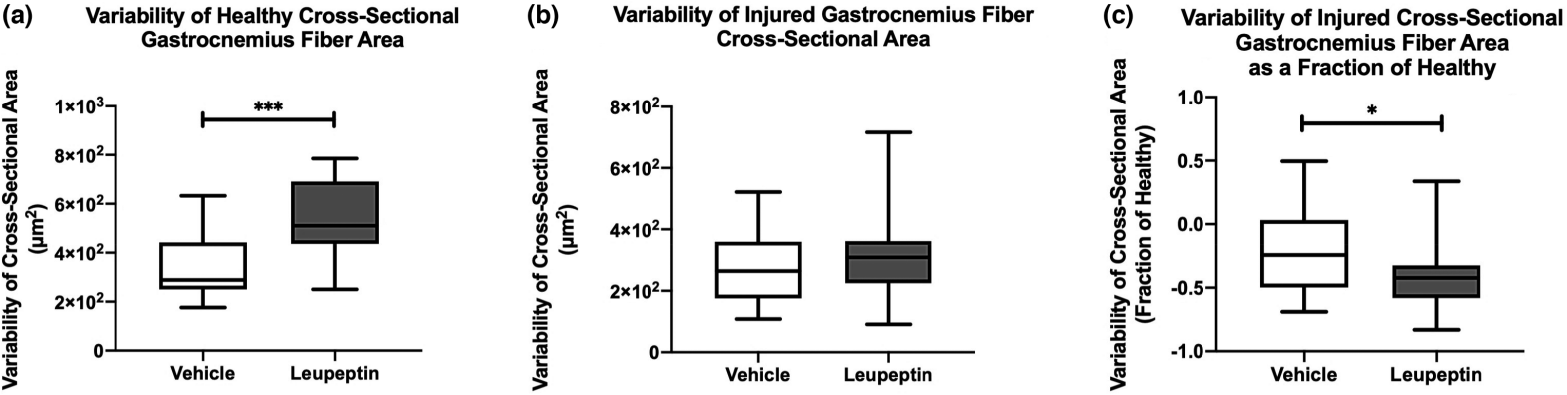
(a) Variability in healthy gastrocnemius muscle fiber cross‐sectional areas are larger after leupeptin treatment than vehicle treatment (*n* = 5). *p* < 0.001. Mann–Whitney test. (b) Variability in injured gastrocnemius muscle fiber cross‐sectional areas are not different after leupeptin treatment or vehicle treatment (*n* = 5) (c) variability in injured gastrocnemius muscle fiber cross‐sectional areas is less after leupeptin treatment or vehicle treatment after normalization to healthy muscle fiber cross‐sectional area variability (*n* = 5). *p* < 0.05. Mann–Whitney test.

The variability in cross‐sectional muscle areas medians of the healthy gastrocnemius muscle fibers were larger in leupeptin‐treated animals than vehicle‐treated controls, 510.3 vs. 289.1 μm (Pedowitz, 1991), respectively (*p* = 0.0003) (Figure 5a). The medians of the cross‐sectional area variabilities of injured gastrocnemius muscle fibers were not different between leupeptin‐treated animals and vehicle‐treated controls, 309.2 vs. 264.1 μm (Pedowitz, 1991), respectively (*p* = 0.3051) (Figure 5b). When normalized as a fraction of contralateral, healthy muscle fiber areas, the medians of the cross‐sectional areas of injured gastrocnemius muscle fibers were less in leupeptin‐treated animals than vehicle‐treated controls, −0.42 vs. −0.24, respectively (*p* = 0.0249) (Figure 5c).

### 3.5 Cross‐sectional areas of sciatic nerves

**FIGURE 6:**
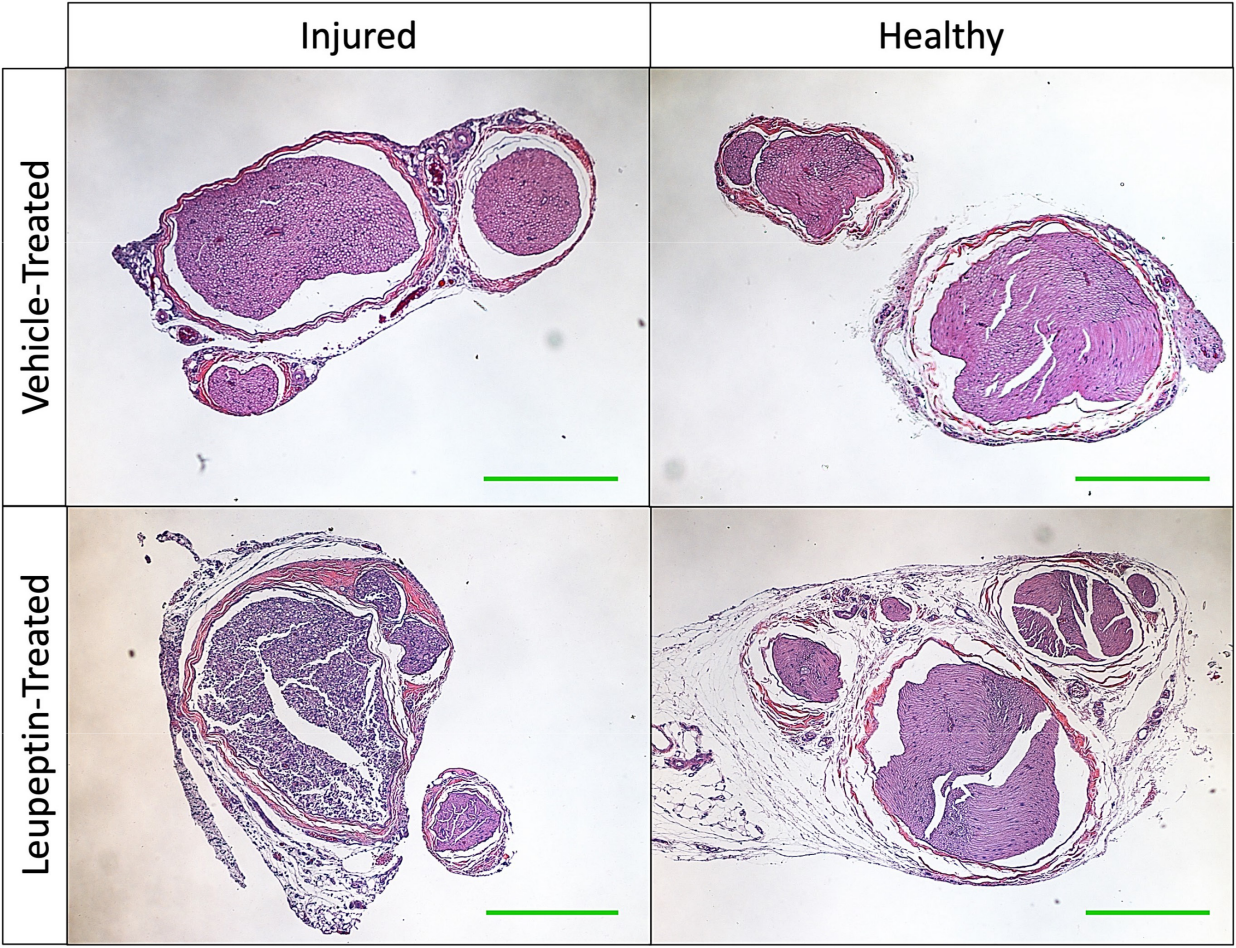
5 μm cross‐sections of injured and contralateral healthy sciatic nerve stained with hematoxylin and eosin for representative animals treated with vehicle or leupeptin 2 weeks after two‐hour blood flow occlusion. Green size‐bars indicate 500 μm.

**FIGURE 7:**
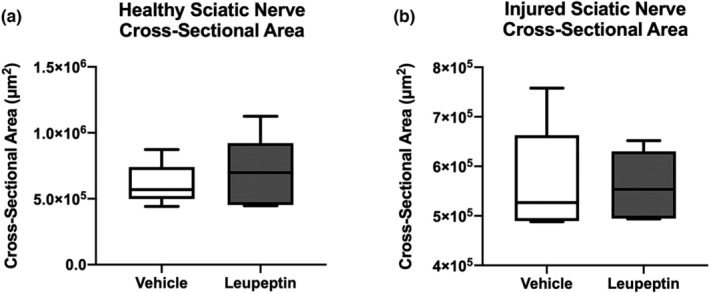
(a) Healthy sciatic nerve cross‐sectional areas not different after leupeptin treatment than vehicle treatment (*n* = 5). (b) Injured sciatic nerve cross‐sectional areas are not different after leupeptin treatment or vehicle treatment (*n* = 5).

Representative cross‐sections of injured and contralateral healthy sciatic nerve 2 weeks after injury, with vehicle and leupeptin treatment, respectively, are presented in Figure 6. The average of the cross‐sectional areas of healthy sciatic nerves were not different in leupeptin‐treated animals compared to vehicle‐treated controls, 6.9 × 10 (Kilinc et al., 2011) (2.5 × 10^5^) vs. 6.1 × 10^5^ μm^2^ (1.5 × 10^5^), respectively (Figure 7a). The average of the cross‐sectional areas of injured sciatic nerves were not different between leupeptin‐treated animals and vehicle‐treated controls, 5.6 × 10^5^ (6.7 × 10^4^) vs. 5.7 × 10^5^ μm^2^ (1.1 × 10^5^), respectively (Figure 7b).

## 4 DISCUSSION

Ischemia and ischemia–reperfusion injury (IRI) can lead to major problems during heart attacks, organ transplantations, replantation, and in microsurgery and complex reconstructive surgical procedures. Ischemia and IRI induce a complex series of events that can lead to cell death and degeneration of nerves and muscles. Over the past few decades, several different methods of tissue conditioning have been employed to mitigate these adverse effects, such as surgical delay, ischemic preconditioning, use of growth factors, and thermic preconditioning (Krauss et al., 2018). However, no single optimal method has been identified. In our current study, we targeted calpain as it is known to be a major trigger for this cascade of undesired events by treating the rats with the proven calpain inhibitor, leupeptin. We hypothesized that leupeptin could prevent nerve and muscle injury following ischemia at histological and functional levels.

Interestingly, leupeptin aided in increasing muscle fascicles average cross‐sectional area and variability of fiber areas of healthy contralateral muscles, but did not do so for muscle after damage from two‐hour ischemia. This can be seen qualitatively in the representative histological images and graphs summarizing those data. A larger average area is representative of overall, more hypertrophic muscle, while larger variability in areas is indicative of a broader range of muscle fiber types as not all types are of similar cross‐sectional area. This could be because of the uneven and non‐uniform migration of leupeptin to muscle cells. This dose of leupeptin administered resulted in a difference in the weights of healthy contralateral muscles as compared to the control. However, the damaging effects of tourniquet‐induced ischemia could not be overcome by this dose of leupeptin in the injured muscles.

We determined there was no enhancement in the nerve areas following administration of leupeptin. The efficacy of leupeptin (specifically C101, a derivative of leupeptin) was questioned in a study reported by Childers et al. They found that oral administration of C101 in young dogs with golden retriever muscular dystrophy (GRMD) did not improve muscle pathology and weakness compared to the control group (Childers et al., 2011). However, there are other molecules that have been successful in inhibiting calpain and preserving tissue functions. David et al. used a novel calpain peptide inhibitor, cysteic‐leucyl‐argininal (CYLA) and demonstrated successful restoration of retinal function in a rat model of transient retinal ischemia. This peptide was administered systemically following ischemia and was able to cross the blood‐retinal barrier, in contrast to leupeptin that was found to be ineffective (David et al., 2011). Thus, the types of molecules employed, their modes and timings of administration clearly must be accounted for in effective inhibition of calpain.

Another limitation of our study is the lack of measurement of blood flow in the reperfused limb as numerous studies have demonstrated improved perfusion following calpain inhibition in small and large animal models (David et al., 2011; Sabe et al., 2016). For example, Sabe et al. reported that calpain inhibition improved perfusion and increased capillary density in a swine model of chronic myocardial ischemia (Sabe et al., 2016). David et al. found that calpain inhibition reduced retinal cell death and preserved function following ischemia (David et al., 2011). Similarly, calpain inhibitors were found to be effective in promoting the growth of neovessels that were both architecturally and functionally effective in improving vascular perfusion and reducing hypoxia in a mouse model of retinopathy (Hoang et al., 2011) Finally, Bartus et al. identified significant protection against ischemic brain damage in rat models after treatment with AK275 (Z‐Leu–Abu–CONH–CH2CH3) – a calpain inhibitor (Bartus et al., 1994).

Other researchers have used knockout models to study the pharmacological effects of calpain inhibitors. Tonami et al. showed that Calpain‐6 knock‐out mice demonstrate increased skeletal‐muscle development and regeneration (Tonami et al., 2013). Similar positive outcomes were observed in another study conducted by Yu et al., that used calpain‐1 knockout rats and found improvements in locomotor function following contusive spinal cord injury (Yu et al., 2013). Overall, the evidence suggests that repressing calpains through transgenic models or pharmaceutical inhibitors results in equivalent effects (Tian et al., 2020; Wang et al., 2020).

Several factors could have played a role in our reported lower than expected efficacy of leupeptin. We believe that more frequent leupeptin injections, along with a higher concentration may have resulted in significant improvements. The timing of leupeptin administration could also play a role as injecting it before tourniquet application could lead to significant improvements in rats. Future studies will be designed to specifically answer these questions.

In summary, leupeptin was able to induce growth of contralateral healthy muscles as evidenced by statistically significant increases in fascicle area as compared to the control group. However, no improvements with the current dosage regimen were observed with respect to the gait, nerve tissue, and muscle tissue recovery after ischemic injury.

### AUTHOR CONTRIBUTION

MG – Conceptualization, Data curation, Formal Analysis, Investigation, Visualization Writing – review & editing. KI ‐ Investigation, Data curation, Visualization. INM ‐ Investigation, Visualization. GS – Conceptualization, Data curation, Formal Analysis, Investigation, Visualization, Project administration, Supervision Writing – review & editing. SUK ‐ Investigation, Visualization. DTB – Investigation, Visualization. ABD ‐ Conceptualization, Investigation, Visualization. DEK ‐ Conceptualization, Data curation, Formal Analysis, Funding acquisition, Methodology, Project administration, Resources, Supervision, Validation, Visualization, Writing – review & editing.

### FUNDING INFORMATION

Funding for this study was kindly provided by the Department of Orthopedics and Rehabilitation and The Department of Surgery at Stony Brook University.

### CONFLICT OF INTEREST

None of the authors have any conflicts of interest.

## ETHICS APPROVAL STATEMENT

This study was approved the Stony Brook University Institutional Animal Care and Use Committee prior to initiation.
